# Robotic versus open pancreatoduodenectomy (SPAIN Trial): protocol for a multicenter randomized controlled trial assessing safety, efficacy and cost-effectiveness

**DOI:** 10.1007/s13304-026-02574-1

**Published:** 2026-06-09

**Authors:** Alberto Carrancho, Danilo Vinci, Fernando Burdio, Patricia Sànchez-Velázquez, Benedetto Ielpo, SPAIN Trial group, Candido Alcazar, Jose Manuel Ramia, Constantino Fondevila, Alberto Garcia-Picazo, Elizabeth Pando, Nair Fernandes, Emilio Vicente, Riccardo Caruso, Valentina Ferri, Maria Alejandra Guerrero-Ortiz, Rosa Maria Jorba, Robert Memba, Fulthon-Frank Vela, Alfredo Escartin-Arias, Santiago Sanchez-Cabus, Victor Molina, David Pacheco, Francisco Javier Tejero-Pintor, Marta Gimeno-Lopez, Amelia J. Hessheimer

**Affiliations:** Hepato-Biliary and Pancreatic Surgery Unit, Department of Surgery, Hospital del Mar, Pompeu Fabra University, Passeig Maritim 25, Barcelona, 08034 Spain

**Keywords:** Robotic, Pancreatoduodenectomy, Cost-effectiveness

## Abstract

Minimally invasive pancreatoduodenectomy (PD), particularly through a robotic approach (RPD), is gaining increasing worldwide acceptance. However, high-quality evidence remains limited and very few randomized controlled trials have directly compared RPD with open PD (OPD). The SPAIN Trial (Spanish Pancreatoduodenectomy: Open vs. Robotic Approach) is designed to generate national evidence on the safety, efficacy, and economic value of RPD compared with the open technique. This is a national, multicentre, randomized, prospective, non-inferiority trial comparing RPD and OPD in high-volume Spanish centres with established robotic pancreatic programs. Adult patients indicated for elective PD due to benign or malignant disease of the pancreatic head, distal bile duct, duodenum, or ampulla of Vater will be randomized in a 2:1 ratio (2RPD:1OPD). The primary endpoint is overall postoperative morbidity within 90 days, assessed by the Comprehensive Complication Index (CCI). Secondary endpoints include intraoperative outcomes (operative time, blood loss, conversion rate), ISGPS-defined pancreatic complications, oncological outcomes (R0 resection, lymph node yield, time to adjuvant therapy), length of stay, readmission rates, and patient-reported quality of life using EQ-5D-5 L, EORTC QLQ-C30, and PAN-26 questionnaires. Cost-effectiveness will be assessed using Quality-Adjusted Life Years (QALYs) and Incremental Cost-Effectiveness Ratios (ICERs) from a healthcare perspective.

*Trial registration*: ClinicalTrials.gov Identifier NCT06981273.

## Background

Minimally invasive pancreatoduodenectomy (PD), particularly through a robotic approach, has gained increasing interest worldwide. However, the available scientific evidence remains limited, with few randomized studies and inconsistent results. To date, only two studies have been published that directly compared the open and robotic approaches [1, 2], one study has included both the robotic and laparoscopic approaches [3], and there is one ongoing trial comparing the open and robotic approaches [4].

Recent contributions from Boggi et al. [5]– [6] have expanded the evidence base, providing comprehensive reviews on the state of the art of robotic PD (RPD) and highlighting both technical advantages and current limitations; furthermore, updated consensus guidelines, such as the REDISCOVER initiative, now provide structured recommendations for borderline and locally advanced pancreatic cancer, underscoring the importance of standardized management algorithms [7].

This trial will contribute significantly to generating robust evidence on the safety, efficacy, and cost-effectiveness of the robotic approach in PD, with direct implications for clinical practice and healthcare decision-making.

## Current evidence

The PD is a highly complex surgical procedure. It involves resection of the main bile duct, gallbladder, part of the stomach, the duodenum up to the first jejunal loop, and the pancreatic head. Following removal of the surgical specimen, it is necessary to restore the continuity of the digestive tract and reestablish communication between the pancreas and bile duct with the intestine by performing at least three anastomoses: a pancreatoenteric anastomosis, a bilio-enteric anastomosis, and a gastrojejunostomy. The proper construction of the pancreatoenteric anastomosis after PD is a critical factor in preventing postoperative complications, such as postoperative pancreatic fistula, and represents one of the most challenging aspects faced by hepato-pancreato-biliary surgeons [8].

PD is indicated for pancreatic head cancer as well as for a wide range of benign and malignant diseases of the pancreas and distal bile duct [8, 9]. Advances in surgical techniques and perioperative care have reduced perioperative mortality of pancreatic resection to less than 5% in high-volume specialized centres [7–9]. However, perioperative morbidity remains high, with rates around 40% [10–13]. Therefore, measures to reduce postoperative complications are urgently needed. Given the technical complexity, open PD (OPD) remains the most used standard technique.

Minimally invasive surgery (laparoscopic and robotic) has been shown to reduce postoperative morbidity in a wide range of surgical procedures [5, 14, 15]. Laparoscopic PD was introduced in 1994, and RPD in 2003 [13]. Several studies, with small sample sizes, non-randomized designs, mostly retrospective and single-centre, as well as case series, have investigated minimally invasive approaches and compared them to OPD, yielding inconsistent results [2, 16, 17]. However, most of these studies involve the laparoscopic approach, which presents significant limitations due to the restricted movement of laparoscopic instruments. In contrast, the robotic system allows a greater range of motion with enhanced three-dimensional vision, which may facilitate PD and potentially improve outcomes, particularly in terms of functional recovery, an area still understudied in surgical clinical trials [5].

Moreover, evidence on specific surgical scenarios, such as the impact of tumor proximity to landmark vessels in minimally invasive-RAMPS, illustrates how robotic surgery may facilitate technically demanding procedures [16].

The RPD is beginning to be integrated into clinical practice for selected patients and in high-volume centres. Several international randomized controlled trials conducted in specialized centres, such as the ongoing results of DIPLOMA-2 trial, have shown shorter hospital stays, reduced perioperative blood loss, and complication rates comparable to those of OPD [3].

### The rationale for the study

Currently, there are 180 hospital centres in Spain equipped with robotic surgical systems. The adoption of RPD is steadily increasing, driven by its advantages such as enhanced ergonomics and three-dimensional visualization. Several international programs have successfully integrated this approach. However, only two randomized controlled trials, one from Germany (EUROPA trial) and one from China (LIU trial), have directly compared RPD with the OPD approach, demonstrating non-inferiority of RPD [1, 2].

Despite these findings, outcome data on RPD are lacking in Spain. As more surgeons begin adopting RPD, it is essential to establish whether this technique provides outcomes at least equivalent to, if not better than, those of the conventional open approach, particularly in the Spanish context, where the number of centres performing RPD continues to grow.

To address this gap, a multicentre randomized controlled trial in high-volume Spanish centres was designed to evaluate the safety and cost-effectiveness of robotic pancreatoduodenectomy, complementing existing randomized evidence [1, 2].

## Aims of the study

### Primary aim of the study

This clinical trial aims to demonstrate the non-inferiority of two different surgical approaches: RPD and OPD, with respect to postoperative morbidity within 90 days of surgery, as measured by the Comprehensive Complication Index (CCI^®^) [19].

## Secondary aims of the study

Secondary objectives include intraoperative variables (including blood loss, operative time, and conversion rate), postoperative morbidity (including pancreatic surgery-specific complications according to ISGPS definitions [13, 14], oncological outcomes in cases of malignancy (including radicality of resection, number of lymph nodes retrieved, and time to initiation of adjuvant chemotherapy), healthcare resource utilization (including length of hospital stay, readmission rates, ICU stay), and quality of life and postoperative recovery evaluated with QALYs [20–23].

## Study design

National, multicentre, randomized, prospective, parallel-group clinical trial comparing patients undergoing PD for any indication.

Patients will be randomized into one of the following groups:

- ***Group 1***: Robotic Pancreaticoduodenectomy (RPD).
- ***Group 2***: Open Pancreaticoduodenectomy (OPD).

The trial will be conducted in centres with a minimum experience of at least 30 RPD cases.

### Study population and sample size

This is a non-inferiority study. According to previous research, a clinically relevant mean difference for the CCI is 10, while the average standard deviation is approximately 20 in major abdominal surgeries [20]. A total of 120 patients will need to be enrolled in the trial with a 2:1 ratio (80 in the robotic group and 40 in the open group), accounting for a 5% loss rate, with an alpha error of 5% and beta error of 20%.

A 2:1 randomization was chosen to avoid disruption of the ongoing learning curve in participating centres and to ensure sufficient inclusion of open procedures for robust comparative analysis.

## Centre and patient inclusion/exclusion criteria

### Centre inclusion criteria

- The centre and the lead surgeon must have adequate experience, performing at least 30 RPD cases.
- Approval of local ethical committee.

### Patient inclusion criteria

- Minimum age of 18 years.
- Indication for elective PD due to a tumour located in the head of the pancreas, distal bile duct, duodenum, or ampulla of Vater, including both benign and malignant tumours.
- Both RPD and OPD must be technically feasible for radical resection, as determined by the local multidisciplinary treatment team.
- Preoperative multiphase CT scan without signs of vascular involvement (3D reconstruction optional), or in cases of suspected malignancy: CT must be no older than 30 days, and is required to rule out vascular infiltration and metastatic disease, which are exclusion criterion.
- Deemed fit for minimally invasive PD by both the surgeon and anaesthesiologist.
- Written informed consent obtained.

### Patient exclusion criteria

- A second cancer requiring resection during the same procedure.
- Chronic pancreatitis as the surgical indication (including groove pancreatitis).
- Any vascular involvement (portal vein, superior mesenteric vein, superior mesenteric artery, celiac artery, or hepatic artery).
- Pregnancy.
- Participation in another study that may interfere with study outcomes.
- Inability or unwillingness to complete quality of life questionnaires.
- High-risk patients with severe comorbidities (ASA IV) as defined by the American Society of Anaesthesiologists.
- Metastatic disease.

### Criteria for subject withdrawal from the study

A patient may be withdrawn from the study for any of the following reasons:

1. Voluntary withdrawal by the patient, including withdrawal of consent. Patients have the right to leave the study at any time.
2. Investigator decision to withdraw the patient for any reason deemed necessary, including retrospective discovery of ineligibility.

Patients who withdraw will continue to receive standard medical care according to local clinical practice.

## Data collection

### Clinical data

Clinicians will be asked to record medical data related to surgery (including operative equipment, skin-to-skin operating time, blood loss, surgical instruments used, and the number of surgeons), hospitalization (intensive care unit, high-dependency unit, general ward), and any complications with their corresponding management (interventions, additional imaging, or extended hospitalization).

Data will be entered into the electronic case report form (eCRF) (REDCap; Research Electronic Data Capture).

Complications will be classified according to the Clavien–Dindo classification of surgical complications [26] and documented as either adverse events or serious adverse events.

Overall morbidity will be quantified using the Comprehensive Complication Index (CCI) [19].

The 95% confidence intervals (CIs) for the difference in means between the two groups will be presented as the primary result. As the CCI follows an approximately normal distribution, the 95% CI will be calculated using the normal distribution. Additional descriptive statistics will be expressed as mean and standard deviation.

Missing data will be managed according to the underlying cause. If the missingness is directly or indirectly related to the treatment, it may introduce bias even in an intention-to-treat analysis. In such cases, missing data will be reported in the final study report, and in the intention-to-treat analysis they will be handled using the worst-case scenario method (classified as failure).

## Data completeness

### Questionnaires

All participants will be required to provide additional informed consent to complete the validated EuroQol EQ-5D-5 L™ questionnaire (official Spanish version). They will be asked to assess five dimensions of health status using the EQ-5D™: mobility, self-care, usual activities, pain/discomfort, and mood (anxiety/depression). This instrument is considered the most representative quality-of-life tool for cost-effectiveness analyses [27]. Questionnaires will be administered at baseline (inclusion), and at one and three months postoperatively, delivered by mail, electronically, or in person, according to the participant’s preference.

### Cost data

Cost data will be obtained from the Spanish Hospital Costs Network (Red Española de Costes Hospitalarios, RECH; https://www.rechosp.org/rech/faces/en/jsf/index.jsp). RECH is an official Spanish initiative aimed at developing a comprehensive database to provide reliable per-patient hospital care cost information. This database consolidates cost data from multiple institutions within the Spanish Healthcare System.

The analysis will include all direct hospital costs, excluding expenses related to the acquisition or maintenance of the robotic system. Pre-admission screening charges will not be considered, as they are identical for both surgical approaches. Costs associated with any hospital readmission will be added to the total hospital expenditure.

A 3% annual discount rate will be applied to both costs and QALYs, in accordance with health economic guidelines [27]. All costs will be expressed in Euros, using the 2025–2026 exchange rate.

## Auditing

Independent, qualified investigators will be responsible for monitoring the study. Data entered in the eCRF are periodically monitored and once monitoring has been completed, visits will be designated as “complete data.” The timing of monitoring visits will be scheduled in advance in agreement with participating centres. Data completeness, accuracy, and plausibility will be assessed at the time of entry through programmed edit checks and validation procedures that generate queries. The completed eCRF must be reviewed and signed by the investigator identified in the trial protocol or by an authorized sub-investigator. The investigator, or their designated representative, is required to complete the eCRF promptly after data collection and to respond to, clarify, or resolve any outstanding queries.

### Safety reporting

Safety reporting will be based on local protocols for every centre separately.

Adverse events are defined as any undesirable experience occurring to a patient during the study, whether considered related to the surgical procedure. All (serious) adverse events should be collected up to 90 days postoperative.

Serious adverse events (SAE) are defined as following:

- Reoperation.
- Death.

Local investigators are responsible for the reporting of SAE’s within 24 h upon their knowledge to the central trial coordinators. Furthermore, they should follow local practice regarding SAE reporting in their own centre.

### Adverse events (AEs)

In the context of pancreatic surgery, an adverse surgical event is defined as any unintended complication or harm directly attributable to the surgical procedure or its perioperative management, and not to the patient’s underlying disease. This includes postoperative pancreatic fistula (POPF) [17], delayed gastric emptying (DGE) [17], post-pancreatectomy haemorrhage (PPH) [18], biliary or anastomotic leaks, intra-abdominal collections, surgical site infections, reoperations, unplanned invasive procedures, prolonged hospitalization, or death. Adverse events will be classified according to the International Study Group of Pancreatic Surgery (ISGPS) definitions and graded using the Clavien–Dindo classification [17, 19, 26], with grades III–V considered serious adverse surgical events.

### Follow-up of adverse events

Adverse events (AEs) will be monitored for a minimum of 90 days following treatment. Each AE will be followed until resolution or until a stable clinical condition is achieved. Depending on the nature of the event, additional diagnostic tests, therapeutic procedures, or referral to a general practitioner or specialist may be required. Common postoperative complications in pancreatic surgery, such as postoperative pancreatic fistula (POPF) and post-pancreatectomy haemorrhage (PPH), will not be systematically reported as AEs, as they are frequent in this patient population. All other adverse events will be documented until the end of the study, in accordance with the study protocol.

### Data safety monitoring board

An independent Data Safety Monitoring Board (DSMB) will be established to oversee study safety parameters for all participants. The DSMB will convene after the inclusion of every 25 patients once 90-day follow-up has been completed for this cohorts. Meetings may be held via telephone or video conference. The board will consist of two independent statisticians, one independent oncologist, and two independent foreign surgeons. One clinician will serve as DSMB chairperson and another as secretary. Meeting minutes will be prepared by the study coordinator and forwarded to the study sponsor. The DSMB will remain unblinded and will have full access to all serious adverse events (SAEs). If necessary, the DSMB may request comprehensive reports on specific study outcomes.

### Study monitoring

In centres where monitoring is required, a Clinical Research Associate will oversee study conduct. Monitoring visits will be arranged in agreement with the sponsor and scheduled at mutually convenient times, occurring periodically at a frequency deemed appropriate. These visits will assess study progress, verify that participants’ rights and well-being are safeguarded, confirm that the reported data are accurate, complete, and verifiable against source documents, and ensure compliance with the approved protocol, amendments, Good Clinical Practice (GCP), and applicable national regulations. During monitoring, essential study documents (e.g., regulatory files, case report forms, source documents, informed consent forms) will be reviewed, and discussions with investigators will be held regarding study conduct. Investigators are expected to be available to facilitate access to study records and to address, resolve, and document any discrepancies identified.

### Statistical method

Primary and secondary endpoint data from all randomized patients will be analysed according to the intention-to-treat principle, based on initial treatment assignment. Additional per-protocol and as-treated analyses will also be performed. The primary endpoint will be tested for non-inferiority using either Chi-square or Fisher’s exact test, as appropriate. Secondary outcomes will be analysed as follows: normally distributed continuous variables with independent samples t-tests (expressed as means ± standard deviations); non-normally distributed continuous variables with Mann–Whitney U tests (medians and interquartile ranges); and categorical variables with Chi-square or Fisher’s exact tests (proportions). Statistical significance will be set at a two-tailed p-value < 0.05.

Time-to-event outcomes (e.g., survival) will be analysed using Kaplan–Meier methods, with Cox regression used to identify predictors of postoperative survival. Variables with *p* < 0.1 in univariable analyses will be entered into multivariable Cox regression. Multivariable models will also be applied to determine predictors of key outcomes such as major complications, postoperative pancreatic fistula, R0 resection, receipt of adjuvant chemotherapy, and hospital readmission. Sensitivity analyses (best- and worst-case scenarios) will be performed for patients lost to follow-up.

Primary endpoint data (Comprehensive Complication Index, CCI^®^) will be actively monitored by the trial coordinator to ensure completeness. Any missing data must be explained to the project leader. If the primary endpoint is missing, mean imputation within the treatment group will be applied. Sensitivity analyses will include imputing the lowest and highest values of the treatment group to model best- and worst-case scenarios. Non-inferiority analyses will also be conducted for cumulative morbidity (CCI, based on Clavien–Dindo classification).

## Methods for minimizing bias

The number of patients screened, the number included, and those analysed will be reported, and differences will be explained. In addition, the patient flow and the Consolidated Standards of Reporting Trials (CONSORT) flowchart will be reported in the final analysis [25].

The quality of life questionnaires are sent directly to patients at predefined time points without investigator mediation, thereby minimizing investigator influence on data recording and interpretation.

In addition, analysis will be stratified according to each surgeon’s previous experience before starting the study, type of hospital (public, university) and total number of beds.

## Interim analysis

An interim analysis of the primary outcome will be performed after inclusion of 50% of the sample.

### Cost-effectiveness analysis

The economic evaluation of RPD versus OPD will be conducted as a cost-effectiveness analysis from a health care perspective, with a 2-year time horizon. The primary outcome will be the cost per quality-adjusted life year (QALY), expressed as the incremental cost-effectiveness ratio (ICER).

Analyses will follow the intention-to-treat principle.

Relevant resources identified for this evaluation include direct medical care costs, such as length of hospital stay (including intensive care unit stays), number and type of diagnostic and therapeutic procedures during the 90 postoperative day period. Resource use will be captured through eCRF, patient questionnaires, and hospital information systems. Postoperative complications will be recorded from the 90-day follow-up questionnaire. Health-related quality of life will be assessed using the EQ-5D-5 L instrument at 2 weeks, 3 months, 6 months, 1-year, 2-years after surgery.

Resource valuation will be based on the Spanish manual for costing in health care research, supplemented with data from hospital administrative sources.

Costs will be expressed in 2026 Euros, and unit costs from different calendar years will be standardized using annual consumer price indices.

### Quality of life

Validated instruments, including the EORTC QLQ-C30 and the pancreas-specific PAN-26 questionnaire, will be used to assess health status and quality of life over time. Patients will be asked to complete these questionnaires at baseline, and at 1 month, 3 months, 6 months, and 36 months postoperatively. Questionnaires will be distributed centrally, electronically via email and automatically registered in the eCRF.

## Ethics and dissemination

The study has been registered in ClinicalTrials.gov (NCT06981273).

The Local Ethical Committee of the Coordinating Centre (Parc Salut Mar Hospital, Barcelona) has approved this study with the code: 2025/11,963/I, and institutional review boards of the participating centres will be requested to provide their approval.

Furthermore, the study is approved by the Spanish Surgical Association committee which funded this study with its budget.

The study will be conducted in compliance with the Declaration of Helsinki and Good Clinical Practice (GCP) guidelines [20]. The protocols and any amendments will be submitted for approval to the appropriate independent ethics committees at each participating centre.

Written informed consent will be obtained from all participants prior to inclusion in the study.

The results of the study will be disseminated through peer-reviewed journals, presentations at international conferences, and updates to clinical guidelines.

The authors will ensure open access publication where possible and follow CONSORT reporting standards [25].

## Study limitation

This trial is conducted in high-volume centres with established expertise in pancreatic and robotic surgery and excludes patients requiring vascular resections or presenting with advanced disease or severe comorbidities. Although these design choices may limit immediate generalizability to more complex real-world settings, they were intentionally adopted to ensure patient safety, procedural standardization, and methodological rigor in the initial randomized evaluation of robotic pancreatoduodenectomy. As with most randomized surgical trials, the findings should be interpreted within this context and regarded as a foundational step upon which future pragmatic studies and real-world registry analyses may build.
